# 100 days with scans of the same Catphan phantom on the same CT scanner

**DOI:** 10.1002/acm2.12186

**Published:** 2017-09-18

**Authors:** Ellen Husby, Elisabeth D Svendsen, Hilde K Andersen, Anne Catrine T Martinsen

**Affiliations:** ^1^ Department of Diagnostic Physics Oslo University Hospital Oslo Norway; ^2^ Department of Diagnostic Imaging Akershus University Hospital Akershus Norway; ^3^ Department of Physics University of Oslo Oslo Norway

**Keywords:** Catphan, computed tomography, image quality, phantom, quality control

## Abstract

Quality control (QC) of CT scanners is important to evaluate image quality and radiation dose. Different QC phantoms for testing image quality parameters on CT are commercially available, and Catphan phantoms are widely used for this purpose. More data from measured image quality parameters on CT are necessary to assess test methods, tolerance levels, and test frequencies. The aim of this study was to evaluate the stability of essential image quality parameters for axial and helical scans on one CT scanner over time. A Catphan 600 phantom was scanned on a Philips Ingenuity CT scanner for 100 days over a period of 6 months. At each day of testing, one helical scan covering the entire phantom and four axial scans covering four different modules in the phantom were performed. All images were uploaded into Image Owl for automatic analysis of CT numbers, modular transfer function (MTF), low‐contrast resolution, noise, and uniformity. In general, the different image quality parameters for both scan techniques were stable over time compared to given tolerance levels. Average measured CT numbers differed between axial and helical scans, while MTF was almost identical for helical and axial scans. Axial scans had better low‐contrast resolution and less noise than helical scans. The uniformity was relatively similar for axial and helical scans. Most standard deviations of measured values were larger for helical scans compared to axial scans. Test results in this study were stable over time for both scan techniques, but further studies on different CT scanners are required to confirm that this also holds true for other systems.

## 1 INTRODUCTION

Quality control of parameters influencing image quality and radiation dose in CT are mandatory in several countries due to regulations.^1^, ^2^ Several QC phantoms are available for image quality testing of CT scanners, ranging from vendor specific phantoms to widely used commercial phantoms like Catphan (The Phantom Laboratory, Salem NY, USA).^3^, ^4^, ^5^, ^6^, ^7^ Publications by international organizations like the International Electrotechnical Commission (IEC) and Institute of Physics and Engineering in Medicine (IPEM) provide specific recommendations related to test methods, test frequencies, and tolerance levels for different QC tests in CT.^8^, ^9^, ^10^ Tolerance levels should be wide enough so that only serious drifting or sudden errors are detected and not clinically irrelevant daily fluctuations. The recommended test frequency should aim to balance between being frequent enough to ensure adequate image quality, while infrequent enough to reduce time spent by the staff and avoid unnecessary interference with clinical work.^6^, ^7^

CT numbers, spatial resolution, low‐contrast resolution, noise, and uniformity are important image quality parameters in QC of CT systems.^5^, ^11^ The CT number describes the x‐ray attenuation characteristics of the material scanned relative to that of water.^11^ Spatial resolution in CT refers to the ability to differentiate small high‐contrast objects in an image.^5^ In the scan plane, the spatial resolution can be described by the MTF.^11^ Spatial resolution is important for example in visualizing iodine‐enhanced vessels or small bone structures.^4^ The low‐contrast resolution refers to the ability to differentiate objects with slightly different density from the background.^5^, ^11^ Clinically, it is important for the ability to distinguish one soft tissue from another.^12^ The noise is the standard deviation of a sample of CT numbers within a region of interest (ROI) in a homogenous part of a CT image.^11^ To reveal beam hardening and cupping artifacts in CT images, uniformity is tested. Uniformity is a measure of the capability of the CT scanner to produce a uniform picture of a homogenous material.^5^, ^7^

Published guidelines are mainly based on image quality parameters evaluated from phantom images acquired in axial scanning mode.^8^, ^9^, ^10^, ^11^, ^13^ However, in clinical practice, helical scans are mostly used based on advantages such as reduction of motion artifacts, scan time, and partial volume effects.^12^ Automatic evaluation of QC images has the advantage of being observer independent and may also be time efficient. Storing of QC data in databases is a valuable tool to allow more in‐depth analysis of failure rates of different test, changes that occur during the lifetime of the equipment and also to compare CT scanners. New developments in CT scanner technology, including important features that affect image quality, are rapidly emerging. It is therefore essential to update methods for QC to make sure the most clinically relevant parameters are being tested.

Discussions on optimal testing methods, tolerance levels, and test frequencies are ongoing in the scientific environment.^2^, ^3^, ^4^, ^5^, ^6^, ^7^ More data from frequent measurements over time provide valuable insight into error rates and day to day fluctuations for the different tests. Together with exploring different test methods, this will help establishing better QC routines for CT scanners. Information on day to day fluctuations of measured image quality parameters is available in the literature, but to our knowledge no study has been performed with Catphan which is widely used by medical physicists.^6^, ^7^ Studies using both axial and helical scan technique for QC are also available, but to our knowledge no systematic comparison of results from the different techniques has been carried out.^4^, ^6^ Data from repeated helical and axial scans on the same CT and Catphan are therefore interesting to (a) compare helical and axial scan techniques and (b) to evaluate fluctuations in measured parameters from a Catphan.

The aim of this study was to evaluate the stability of essential image quality parameters for axial and helical scans on one CT scanner over time.

## 2 METHODS

The same Catphan 600 phantom was scanned on a Philips Ingenuity CT (Philips Medical Systems, Best, Netherlands) for several days over a period of 6 months, resulting in a total of 100 scans. The CT scanner had been in clinical use for about one and a half years when this study was initiated. The workload on the CT scanner before and during this study was about 20 patients per day, 4–5 days a week. Air calibration was performed on the same day before initiating the scans. The phantom was accurately positioned by matching the markings on the outside of the phantom with the coronal, axial, and sagittal lasers on the CT scanner. The accuracy of the lasers was established before initiation and controlled during the course of the study. No changes to the lasers were made during this study. The same specialized CT radiographer performed the majority of the scans, while the same medical physicist performed the scan in case of absence. To ensure that identical scan parameters were used each time, scan protocols were saved on the CT scanner. One helical scan and four axial scans were performed each time. The helical scan covered the entire Catphan. Center positions for the axial scans were at positions 0, −80, −110, and −160 mm along the z‐axis, corresponding respectively to CTP404 sensitometry module (CT numbers), CTP528 high‐resolution module (MTF), CTP515 low‐contrast resolution module, and CTP486 uniformity module (noise and uniformity). Tube current modulation and iterative reconstruction were not applied for any of the scans. The scan protocol was not based on a clinical exam protocol. The helical scan protocol used in this study was developed for standardized acceptance and annual testing, and is used by more than 30 different radiological departments in Norway. The axial scan protocol was designed to be as similar as possible to the helical scan protocol. Scan parameters used for helical and axial scans are presented in Table 1.

**Table 1:** Scan parameters used for helical and axial scans of Catphan 600 phantom

	Tube voltage [kV]	Rotation time [s]	Tube current [mA]	Pitch	Collimation	Slice width [mm]	Kernel	Display field of view (DFOV)	Matrix	CTDI_vol_ [mGy]
Helical scan	120	0.75	324	0.797	64 × 0.625	3	Standard (B)	210	512	20
Axial scans	120	0.75	407	‐	64 × 0.625	3	Standard (B)	210	512	20

The images were uploaded into Image Owl (Image Owl, Inc., Greenwich NY, USA), where analyses of CT numbers, MTF, low‐contrast resolution, noise, and uniformity were performed automatically. Image Owl was also used to group the results from the different tests and dates in to trends. These trend data were then transferred to excel for further analysis. Statistical analysis was performed in IBM SPSS Statistics version 23.0 (IBM Corp, Armonk NY, USA). Linear regression was performed to establish if there were any trends in the data, with the measured values (CT number, MTF, noise, and uniformity) as the dependent variable and the day of scan as the independent variable. The absolute value of the slope of the regression line indicates the rate of change over time. Levene's test was performed to determine if there was a statistically significant difference in variance between helical and axial scans (*P* < 0.05).

### 2.A CT numbers

The sensitometry module (CTP404) of Catphan 600 contains inserts made of air, polymethylpentene (PMP), low‐density polyethylene (LDPE), polystyrene, acrylic, delrin, and teflon. Image Owl automatically positioned ROIs within the different materials for the measurements of CT numbers in Hounsfield units [HU].

### 2.B MTF

The high‐resolution module (CTP528) contains a lower bead point source that was used for evaluation of MTF and the calculation of critical frequency values [cycles/cm] for 50% and 10% of MTF.

### 2.C Low‐contrast resolution

The low‐contrast module (CTP515) contains supra‐slice targets, which are cylindrical objects with diameters of 2–15 mm and having contrast levels of 0.3%, 0.5%, and 1.0%. It also has sub‐slice targets, with z‐axis dimensions of 3, 5, and 7 mm at 1.0% contrast, centered in the z‐dimension of the module. In this study, the supra‐slice targets were used for the low‐contrast evaluation.

### 2.D Noise

The image uniformity module (CTP486) is cast from a uniform material with typical CT number 5–18 HU. The noise was measured in Image Owl as the standard deviation of CT numbers in a central ROI with a diameter 40% of the diameter of the uniformity module.

### 2.E Uniformity

**Figure 1:**
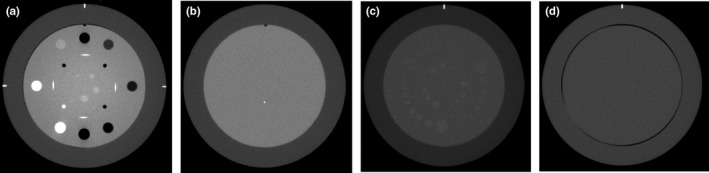
CT images from left to right of (a) the sensitometry module (CTP404), (b) the high‐resolution module (CTP528), (c) the low‐contrast module (CTP515), and (d) the uniformity module (CTP486).

Uniformity was measured from upper, right, lower, left, and central regions ROIs in the same module (CTP486) as noise. The rim of the peripheral ROIs was located 1 cm from the module border. Image Owl calculated the absolute difference between mean CT number in four ROIs in the periphery and the mean CT number in a ROI in the central region. The maximum difference between the mean value of the center ROI and any of the four peripheral ROIs was used to describe the uniformity for each day of scan. Figure 1 displays CT images of the relevant modules from Catphan 600.

## 3 RESULTS

### 3.A CT numbers

Average values, maximum deviation from average values, absolute difference between minimum and maximum values (range), and standard deviations (*σ*) of measured CT numbers for each material are presented in Table 2 for both axial and helical scans.

**Table 2:** Average values, absolute value of maximum deviation from average values, range, and standard deviations of CT numbers

	Scan type	Air	PMP	LDPE	Polystyrene	Acrylic	Delrin	Teflon
Average CT number [HU]	Axial	−1005	−174	−81	−23	138	368	985
Average CT number [HU]	Helical	−988	−175	−85	−27	130	354	953
Max deviation [|HU|]	Axial	8	9	7	3	3	5	23
Max deviation [|HU|]	Helical	5	4	5	6	6	8	14
Range [HU]	Axial	11	12	9	5	6	9	33
Range [HU]	Helical	8	8	9	10	11	15	26
*σ* [HU]	Axial	1.9	1.8	1.1	0.9	1.1	1.9	5.2
*σ* [HU]	Helical	1.8	2.3	2.3	2.5	2.7	3.9	6.4

**Figure 2:**
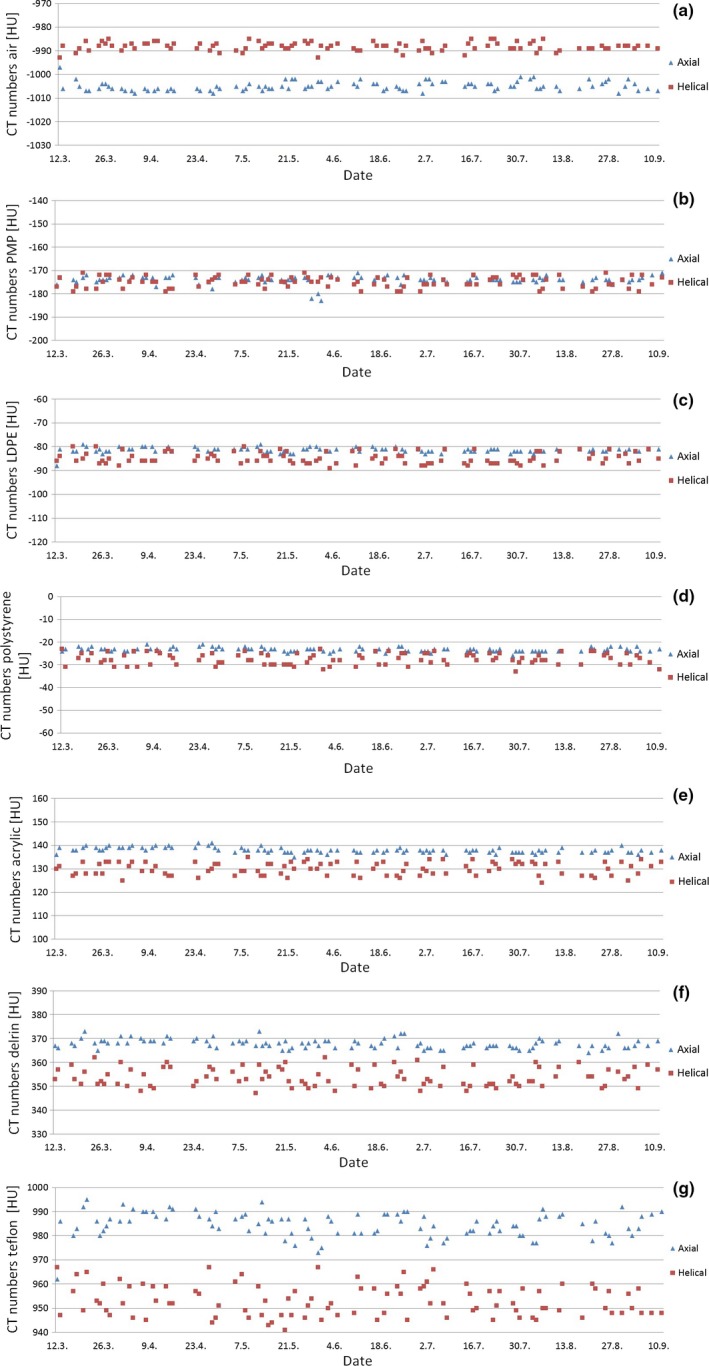
Measured CT numbers for (a) air, (b) PMP, (c) LDPE, (d) polystyrene, (e) acrylic, (f) delrin, and (g) teflon for axial and helical scans.

The axial scans had higher average CT numbers than the helical scans for all materials except air. The difference between axial and helical scans in average CT numbers also increased with increasing density for materials denser than air. The standard deviations of the measured CT numbers were larger for the helical scans than for axial scans for all materials except air. There was a statistically significant difference in variances between axial and helical scan technique for all materials except air. The absolute value of the slopes for the different materials and scan techniques were all <0.017. Figure 2 displays how the measured CT numbers for each of the different materials and scan techniques varies with time.

### 3.B MTF

Average values, maximum deviation from average values, range, and standard deviations (*σ*) of critical frequency values for 50% and 10% of MTF, are presented in Table 3 for axial and helical scans.

**Table 3:** Average values, absolute value of maximum deviation from average values, range, and standard deviation of critical frequency values

	Axial 50% of MTF	Helical 50% of MTF	Axial 10% of MTF	Helical 10% of MTF
Average [cycles/cm]	3.84	3.82	6.59	6.58
Max deviation [|cycles/cm|] and (|%|)	0.13 (3.4%)	0.18 (4.7%)	0.17 (2.6%)	0.24 (3.7%)
Range [cycles/cm]	0.26	0.31	0.26	0.33
(*σ*) [cycles/cm]	0.04	0.05	0.04	0.04

**Figure 3:**
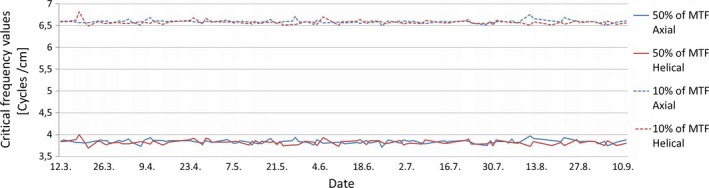
Critical frequency values [cycles/cm] for 50% and 10% of MTF for axial and helical scans.

The average critical frequency values for 50% and 10% of MTF were almost identical for axial and helical scans. The maximum deviation, range, and the standard deviation of critical frequency values were generally smaller for the axial scans than the helical scans, except for the standard deviation for 10% of MTF where there was no difference between the values at the given level of accuracy. There were no statistically significant differences in variances between axial and helical scan technique for 50% or 10% of MTF. The absolute value of the slopes for both 50% and 10% of MTF and both scan techniques were 0.000. Figure 3 displays how critical frequency [cycles/cm] for 50% and 10% of MTF from axial and helical scans varies with time.

### 3.C Low‐contrast resolution

Average values, range, and standard deviations (*σ*) of estimated diameter [mm] of smallest detectable target for each of the three contrast values (1%, 0.5%, and 0.3%) are displayed in Table 4 for axial and helical scans.

**Table 4:** Average values, range, and standard deviation of estimated diameter [mm] of smallest detectable targets

	Axial 1% contrast	Helical 1% contrast	Axial 0.5% contrast	Helical 0.5% contrast	Axial 0.3% contrast	Helical 0.3% contrast
Average [mm]	2.0	2.0	3.0	3.9	5.0	5.9
Range [mm]	0	0	0	1.0	0	1.0
(*σ*) [mm]	0	0	0	0.36	0	0.27

The average low‐contrast resolution was better (smaller detectable targets) for axial scans compared to helical scans for 0.5% and 0.3% contrast. For 1% contrast, the smallest possible target at 2 mm was detected with both scan techniques. The range and standard deviation were larger for helical scans than for axial scans where the measured values did not vary at all over time.

### 3.D Noise

Average values, maximum deviation from average values, range, and standard deviations (*σ*) of measured noise values are presented in Table 5 for axial and helical scans.

**Table 5:** Average values, absolute value of maximum deviation from average values, range, and standard deviation of measured noise

	Axial noise	Helical noise
Average [HU]	4.7	5.3
Max deviation [|HU|] and (|%|)	0.2 (4.1%)	0.2 (3.3%)
Range [HU]	0.3	0.3
(*σ*) [HU]	0.07	0.07

**Figure 4:**
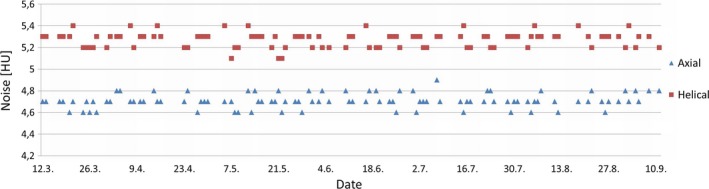
Measured noise for axial and helical scans.

The average measured noise was lower for axial scans than for helical scan, which is consistent with the results from low‐contrast resolution. Range and standard deviation were equal for both axial and helical scans at the given level of accuracy. There was no statistically significant difference in variances between axial and helical scan technique. The absolute values of the slopes for both scan techniques were 0.000. The measured noise for axial and helical scans at different time points are presented in Fig. 4.

### 3.E Uniformity

Average values, maximum deviation from average values, highest measured value, range, and standard deviation (*σ*) of uniformity measurements are presented in Table 6 for both axial and helical scans.

**Table 6:** Average values, absolute value of maximum deviation from average values, highest value, range, and standard deviation for uniformity

	Axial uniformity deviation [HU]	Helical uniformity deviation [HU]
Average [HU]	1.4	1.3
Max deviation [|HU|]	1.1	1.3
Highest value [HU]	2.2	2.6
Range [HU]	1.9	2.2
(*σ*) [HU]	0.3	0.4

**Figure 5:**
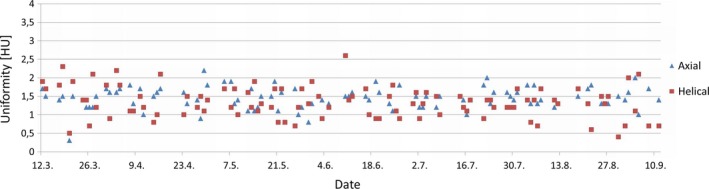
Uniformity for axial and helical scans.

The average value was higher for axial than helical scans, while the highest registered value was higher for the helical scan. Maximum deviation from average values, range, and standard deviation were larger for helical scans than axial scans. There was a statistically significant difference in variances between axial and helical scan techniques. The absolute value for the slopes for the different scan techniques were both <0.002. Uniformity measurements for each day of axial and helical scans are presented in Fig. 5.

## 4 DISCUSSION

### 4.A CT numbers

The measurements of CT numbers are influenced by many factors; effective energy, filtration of the x‐ray tube, and reconstruction algorithms among others.^3^, ^13^ IPEM suggests measuring CT numbers from a range of different density materials at an annual control and measuring water and a high density material (e.g., teflon) daily to weekly. IPEM differentiates between a remedial level at which remedial action needs to be initiated and a suspension level at which it is recommended that the equipment should be removed from clinical use immediately until performance is corrected. For this test, they state a remedial level of ±10 HU for the annual test and a suspension level of ±30 HU for all tests frequencies relative to baseline values.^8^ IEC do not state tolerance levels for measurement of CT numbers from various materials.^9^, ^10^ Measurements of all materials in this study were within ±30 HU from the average value and only teflon were outside ±10 HU for both scan techniques. Measured CT numbers were stable for both scan techniques with no sudden deviations or relevant drifting over time apparent from calculated slopes. However, the day to day fluctuations for teflon in this study indicate that the remedial level of ±10 HU might need to be expanded for teflon.

### 4.B MTF

MTF is most dependent on detector element size, reconstruction matrix, DFOV, scanner geometry, focus size, and reconstruction algorithm.^4^ IPEM suggests a remedial level for yearly constancy control of baseline ±20%, but states no suspension level.^8^ According to IEC, a tolerance level of baseline ±0.5 lp/cm or baseline ±15%, whichever is greater, is given for measurement of the 50% point and 10% point of the MTF curve. IEC recommends a test frequency of at least quarterly.^10^ The measured MTF in this study was very stable over time for both axial and helical scans, where none of the maximum deviations from average values were above 5%. Significant shift in MTF over time is also not expected, even though high resolution scans might show some changes due to focal spot degradation in an aging tube. Software updates like modification of the reconstruction algorithm or hardware changes that affect detector or focus alignment can alter the MTF.^13^ Roa et al evaluated QC data from different CT scanners retrospectively, and also concluded that the spatial resolution did not change much over time.^4^ Calculated slopes showed no apparent trend over time and average values were almost identical between axial and helical scans. Spatial resolution should be comparable between axial and helical scans if the same acquisition parameters are used.^11^

### 4.C Low‐contrast resolution

Low‐contrast resolution is influenced by signal to noise ratio (SNR), the spatial resolution and the reconstruction algorithm.^11^ IPEM do not include low‐contrast resolution in the recommended test regime.^8^ According to IEC, low‐contrast resolution is not a necessary part of acceptance or constancy QC, as if measured noise and CT numbers meet specifications, the low‐contrast resolution is also deemed to meet specifications.^9^, ^10^ The results from our study showed no variations at all in results for axial scans, while helical scans generally had small variations and slightly worse low‐contrast resolution compared to axial scans. This is consistent with less measured noise in axial scans than helical scans.

### 4.D Noise

Noise is a good first‐line measurement, as several different parameters influence noise (like kV, mAs, filtration, slice width, reconstruction algorithm, image filter, and focal spot size), which means that measured deviations can arise from a number of different sources.^13^ IPEM suggest noise measurements to be performed daily to weekly and annually with a remedial level of baseline ±10% and a suspension level of baseline ±25%.^8^ The tolerance level stated by IEC is baseline ±10% or 0.2 HU, whichever is larger, and a test frequency of at least monthly.^10^ The measured noise in this study was very stable over time for both axial and helical scans, none of the maximum deviations from average values were above 5%. The stability of noise found in this study is also consistent with a 2 yr automatic monitoring of QC parameters by Nowik et al Over this period with daily scans, noise levels were only once measured outside their tolerance level of 5% (deviation just above 10%) caused by a barely visible ring artifact.^7^

### 4.E Uniformity

The shaped beam filter, x‐ray tube output, and centering of the object in the beam can influence the uniformity of an image.^7^ IPEM suggest annual control of uniformity with remedial tolerance levels given as the difference in HU between the center and periphery being ±10 HU for head and ±20 HU for body sized water or water equivalent phantoms.^8^ IEC states that the absolute difference between the mean CT number of the central ROI and any of the peripheral ROIs should be compared with given specifications, in the absence of specification the uniformity must not be greater than 4 HU. IEC recommends a test frequency of at least monthly.^9^, ^10^ None of the uniformity results for either scan type was above 4 HU. The study by Nowik et al also tested uniformity in daily scans and none of the measurements went outside a tolerance level of 4 HU and only one measurement went above 2 HU.^7^ IEC also states that the difference between the mean CT number of the central ROI and the outer ROI should not vary by more than 2 HU from those of the baseline.^10^ The maximum deviation from average values was below 2 HU for both scan techniques.

### 4.F General trends and QC routines

In general, the image parameters evaluated in this study were stable over time for both scan techniques. Measured values for both scan techniques fluctuated from day to day with no sudden deviations or relevant drifting over time evident from calculated slopes. As helical scans are mostly used clinically, it is important that they produce images of adequate quality. Axial QC testing will not necessarily reveal deviations apparent in helical images. Helical scanning can also be time efficient compared to axial scanning for QC testing and image analysis. It is expected and apparent in our results that measured values sometimes differs between axial and helical scan techniques. It is therefore important that results should always be compared with baseline values obtained using the same imaging technique. Levene's test concluded that there was a significant difference in variance for the CT number and uniformity test, where the helical scans had larger standard deviations. Helical scans are however stable enough to be used for QC, as this difference is small and not relevant related to the given tolerance levels for these tests.

Acceptance testing (performed at acceptance and when a large change has been made to the scanner, e.g., a tube replacement) and annual QC are typically performed by a medical physicist. At acceptance, QC is performed with vendor protocols to test vendor specifications. In addition, testing both in helical and axial scan mode with fixed protocols is advantageous to establish baseline values. For annual testing, only one helical scan can be performed for image QC. This is supported by the stability of helical scans in this study. Based on the stability over time in this study and others, an annual test frequency for testing of CT numbers, MTF, noise, and uniformity appear sufficient.^4^, ^7^ Low‐contrast resolution may be performed only at acceptance.

Axial scans of a water phantom (preferably large) can be performed by radiographers once a month to visually check for artifacts in addition to measurements of CT number and standard deviation of water. Artifacts are more easily evaluated using axial scan technique as helical scan technique incorporates signal from several detector rows. A study by Nute et al evaluating daily quality control data reported that failure rates were highest for large phantom artifacts (estimated failure rate 6.63 failures/1000 scan days) followed by measured standard deviation in water (estimated failure rate: 5.11 failures/1000 scan days). The measurement in a ROI is fast and would simultaneously give both the CT number and standard deviation in water. The CT number of water, although not with the highest failure rate (estimated failure rate 0.85 failures/1000 scan days), is important due to increased use of quantitative imaging.^6^

Dose measurements and testing of automatic dose modulation should be part of the QC regime of a CT scanner, however, this study only addresses image quality testing, hence, testing of dose and automatic dose modulation is beyond the scope of this study.

## 5 CONCLUSION

The results from this study showed stability over time for all image quality tests for helical and axial scan techniques. However, these results are based on just one CT scanner, and even though other studies have also shown similar tendencies, further studies on different CT scanners are necessary. Additional studies are planned. It is also essential with continued research to develop the most time‐efficient and clinically relevant QC methods and imaging phantoms.

## CONFLICT OF INTEREST

Oslo University Hospital has a research collaboration with Image Owl and The Phantom Laboratory.
